# EEG Resting-state Microstate Dynamics in Children and Adolescents with Avoidant/Restrictive Food Intake Disorder (ARFID)

**DOI:** 10.1007/s10548-025-01149-4

**Published:** 2025-09-30

**Authors:** Kinkini Bhadra, Antony A. Janakiram, Savoia Marco, Nadia Micali, Petra S. Hüppi, Cristina Berchio

**Affiliations:** 1 Division of Development & Growth, Department of Pediatrics, Gynecology and Obstetrics, Faculty of Medicine, University of Geneva, Geneva, Switzerland; 2 Outpatient Unit AliNEA, Division of Child Psychiatry, Department of Women, Children and Adolescents, University Hospitals of Geneva, Geneva, Switzerland; 3 Center for Eating and Feeding Disorders Research, Psychiatric Centre Ballerup, Mental Health Services of the Capital Region of Denmark, Copenhagen, Denmark; 4 Department of Translational Biomedicine and Neuroscience, University of Bari Aldo Moro, Bari, Italy

**Keywords:** ARFID, Eating disorders, EEG, Microstates, Resting-state

## Abstract

Avoidant/Restrictive Food Intake Disorder (ARFID) is a psychiatric condition characterized by extreme food avoidance and limited food intake, leading to nutritional deficiencies, weight loss, and and/or social impairment. Despite its clinical significance, there is a notable lack of research on the neurobiological basis of ARFID. To address this gap, we examined resting-state EEG brain activity in children and adolescents with ARFID, using microstate analysis to capture spatial configurations associated with large-scale brain networks, offering a novel perspective on brain network abnormalities in this population. Eighteen patients with full/subthreshold ARFID (mean age 12.78 ± 3.57) were compared to 18 age- and sex-matched healthy controls (HC) (mean age 14.56 ± 2.85). EEG data were recorded during eyes-closed resting-state and examined using microstate analysis. Four microstate maps were identified (A, B, C, D). Significant differences were observed between groups for Map C, with the ARFID group showing higher values for mean duration compared to the HC group (U = 254, *p* = 0.003). Additionally, a trend towards decreased transition probabilities from microstate B to A (*p* = 0.018) and increased transition probabilities from B to C (*p* = 0.023) were found in ARFID. Source imaging analyses further revealed increased activation in the right posterior cingulate cortex (*p* = 0.005) and decreased activation in the right inferior occipital cortex (*p* = 0.003) in the ARFID group compared to HC. These results suggest distinct patterns of brain activity in children and adolescents with ARFID, particularly involving microstate C, which may reflect atypical Default Mode Network functioning. This study provides preliminary evidence of altered brain network dynamics in ARFID, contributing to a better understanding of its neurobiological basis and offering potential avenues for neurophysiological interventions.

**Supplementary Information:**

The online version contains supplementary material available at 10.1007/s10548-025-01149-4.

## Introduction

Avoidant/Restrictive Food Intake Disorder (ARFID) is a psychiatric disorder characterized by clinically significant nutritional deficits impacting weight/growth, and/or psychosocial dysfunction resulting from significant restriction or avoidance of food intake (Brigham et al. 2018; Fisher et al. 2014; Zimmerman and Fisher 2017). Feeding and eating behaviors observed in ARFID can be associated with different clinical profiles, including aversions to sensory properties of food (such as texture, taste, smell, or appearance), low appetite or limited interest in eating, and/or fear of adverse consequences from eating (e.g., choking) (see Thomas et al. 2017; K. S. Thomas et al. 2025a, b). Although these profiles are not mutually exclusive, they can vary in severity, leading to heterogeneous clinical presentations of ARFID. This behavior may arise from a range of factors, including reactions to traumatic food-related experiences (e.g., fear of choking, gastrointestinal disturbances, hypersensitivity to food texture or olfactory stimuli), comorbid medical conditions (e.g., autism, anxiety) (King et al. 2015; Koomar et al. 2021), and a genetic underpinning (Dinkler et al. 2023). Unlike other eating disorders (ED) such as anorexia nervosa (AN) or bulimia nervosa, ARFID does not involve concerns about body image or a desire to lose weight (Kennedy et al. 2023).

The conceptualization of ARFID has significantly evolved over time. What was once referred to as ‘Selective Eating Disorder’ or ‘Food Avoidance Emotional Disorder’ is now formally recognized as a distinct clinical diagnosis. Its inclusion in both the *Diagnostic and Statistical Manual of Mental Disorders* (5th ed.; DSM–5; American Psychiatric Association, 2013) and the *International Classification of Diseases* (ICD-11, 2019) has solidified its status within the psychiatric community. ARFID affects both children and adults, with prevalence estimates of 5–22% from specialized ED service and 0.3–15.5% from non-clinical samples among children and adolescents (Fisher et al. 2014; Sanchez-Cerezo et al. 2023; Seetharaman and Fields 2020). While the prevalence in adults is less well-defined, emerging research indicates that ARFID remains prevalent in adult populations as well (Kennedy et al. 2023; Nakai et al. 2016). Although some studies suggest that ARFID may be more common in males than in females (Nicholls-Clow et al. 2024), this observed sex difference requires further investigation (Norris et al. 2014). Given the reliance on behavioral assessment for diagnosis, rather than neurobiological markers, identifying individuals at risk and evaluating long-term treatment outcomes present significant challenges (Archibald and Bryant-Waugh 2023; Cara et al. 2023; Seetharaman and Fields 2020).

Despite extensive research into the neurobiology of ED, which suggests altered brain network dynamics (Frank et al. 2019; Mishra et al. 2017), only a few studies have specifically examined the neurobiology of ARFID (Getachew et al. 2021; Kerem et al. 2022; Sailer et al. 2022; Thomas et al. 2017). Thomas et al. (2017) highlighted the neurobiological underpinnings of ARFID, proposing a three-dimensional model of neurobiological alterations in three domains, based on the three main ARFID phenotypes highlighted in DSM-5: (a) sensory perception and sensitivity, resulting in oversensitivity of taste perception, (b) homeostatic appetite regulation, including decreased interest in food, and (c) the negative valence system, characterized by an amplified central nervous system response in anticipation of negative events, such as fear of choking. Supporting elements of this model, Kerem et al. (2022) found hyperactivation in brain regions associated with food anticipation, particularly in the orbitofrontal cortex and insula, in ARFID individuals with higher body weight, which aligns with disrupted appetite regulation. Sailer et al. (2022) also added evidence by observing disruptions in the amygdala-hypothalamic-pituitary-adrenal axis response in ARFID, which may be linked to heightened responses to negative events. Additionally, Getachew et al. (2021) based their hypotheses on prior research in healthy individuals, predicting sex-specific patterns of brain activation in response to high-calorie food cues. They anticipated greater activation in the orbitofrontal cortex and right lateral prefrontal cortex in females and increased activation in the right hippocampus in males. However, these expected sex differences were not observed in individuals with ARFID, suggesting a disruption in typical sex-related food motivation pathways and providing novel insight into the neurobiology of the disorder.

Complementing current neuroimaging findings, electroencephalography (EEG) provides a non-invasive method for recording brain activity with millisecond-level temporal precision, allowing for detailed insights into real-time brain state transitions. Research focusing on resting-state EEG activity during childhood and adolescence in ED has primarily centered on AN (Berchio et al. 2022; Hatch et al. 2011). Increased frontal theta activity has been consistently observed in individuals with AN, both in adolescents (Hatch et al. 2011) and adults (Hestad et al. 2016). Specifically, Hatch et al. (2011) found elevated frontal theta power in adolescents following weight restoration, suggesting that this increase in theta activity could be a stable disorder feature and a potential trait marker. Given that higher theta EEG power during resting state predicts lower cognitive functioning (Tan et al. 2024), these findings underscore the potential for identifying sensitive EEG markers of ED during resting-state conditions.

Microstate analysis for EEG signal evaluation has gained increasing interest in recent years, providing valuable insights into the spatial and temporal dynamics of resting brain networks (Khanna et al. 2015; Michel and Koenig 2018). This data-driven method focuses on quasi-stable scalp topographies that last about 60 to 120 ms (da Cruz et al. 2020; Lehmann et al. 1987; Michel and Koenig 2018). While cluster analysis is commonly employed to identify dominant microstates in spontaneous EEG, the four prototypical maps, microstates A, B, C, and D, have historically been the most investigated (Britz et al. 2010; Koenig et al. 2002; Michel and Koenig 2018; Pascual-Marqui et al. 1995). However, since more recent years have seen the adoption of less constrained clustering approaches in map selection, it is now common to identify between 4 and 7 microstates (see Tarailis et al. 2024). Studies have also linked EEG microstates to resting-state networks observed in fMRI, such as microstate A with the auditory network, microstate B with the visual network (Britz et al. 2010; Custo et al. 2017; Kim et al. 2021; Seitzman et al. 2017), microstate C with salience network or subnetwork of DMN, and microstate D with attentional network (Tarailis et al. 2024).

To the best of our knowledge, no studies have yet investigated resting-state microstates in ARFID. However, this method has been extensively used to study EEG resting-state dynamics in various psychiatric conditions (Bochet et al. 2021; da Cruz et al. 2020; Damborská et al. 2019; Rieger et al. 2016; Thirioux et al. 2023), offering a valuable conceptual and empirical foundation for identifying sensitive neural markers in ARFID as well. For instance, mood and anxiety disorders have been linked with over-engagement of microstates A and B (Chivu et al. 2024). Given the involvement of sensory networks and heightened anxiety in ARFID (Thomas et al. 2025a, b; Zimmerman and Fisher 2017), similar alterations might be expected in this population. Additionally, an increased mean duration of microstate D, linked to executive network functioning, has been reported in individuals with attention deficit hyperactivity disorder (ADHD) (Férat et al. 2022; Luo et al. 2023), suggesting impaired executive control (Berchio et al. 2025). Considering that ARFID is frequently characterized by rigidity in behavior, cognitive inflexibility, and deficits in top-down control (Thomas et al. 2020), it is plausible that disruptions in executive networks may also be reflected in microstate dynamics in this group. Finally, in the only study to date investigating microstates in AN, alterations across multiple microstates were observed, including a reduced occurrence of microstate C, which was linked to heightened focus on internal states (Berchio et al. 2024). Given the emerging evidence of altered interoceptive awareness in ARFID, similar patterns may be expected and warrant empirical investigation. Moreover, in terms of nutritional status, ARFID is also frequently associated with a lower-than-average body mass index (BMI), reflecting the physiological consequences of prolonged dietary restriction (Tack et al. 2022). Given this multifaceted clinical profile, exploring EEG microstate dynamics offers a promising avenue for identifying the neural correlates of physiological dimensions of ARFID.

The primary objective of this study is to describe the spatio-temporal dynamics of large-scale brain networks at rest in ARFID. In addition, we conducted exploratory analyses to examine potential associations between these brain dynamics and overall functioning, including cognitive performance, anxiety levels, and body mass index (BMI), given the limited prior evidence in this area.

Based on Thomas et al.‘s (2017) model, we conducted exploratory analyses to investigate whether individuals with ARFID might exhibit differences in EEG microstate dynamics reflecting core clinical features of the disorder. Specifically, we examined whether heightened sensory sensitivity could be associated with changes in microstates A and B, commonly linked to sensory processing. We also explored potential differences in microstate D, which may reflect disruptions in executive control and attentional processes, possibly related to fear-based avoidance. Finally, we considered microstate C, associated with salience network or DMN activity, potentially relevant to the diminished interest in food often observed in ARFID.

## Materials and methods

### Participants

Eighteen outpatients with ARFID (6 male; mean age 12.78, ± 3.57) were compared to 18 age- and sex-matched healthy controls (HC) (6 male; mean age 14.56, ± 2.85). The ARFID group was recruited from the outpatient unit Alimentation et Nutrition chez l’Enfant et l’Adolescent (ALiNEA) clinic in Geneva, Switzerland. Patients with full (*n* = 17) or subthreshold (*n* = 1) ARFID were included if they met the diagnostic criteria defined in the Diagnostic and Statistical Manual of Mental Disorders, Fifth Edition (DSM-5) (see Supplementary File and Table S1 for further details). HC were recruited through local advertising in youth centers, universities, schools, and social media. Exclusion criteria for all participants were history of head trauma and neurological disorders. For the HC group, additional exclusion criteria included the presence of any history of psychiatric diagnoses such as of autism, and ED. To further rule out psychopathological conditions, participants in the HC group were also administered the Strengths & Difficulties Questionnaire (SDQ), and the cut-off scores were used to exclude significant issues in the domains of emotional problems, conduct problems, hyperactivity, peer relationships, and prosocial behavior (Muris et al. 2003). For the ARFID population, clinical profiles were determined through an anamnesis conducted with the families. In the ARFID sample, the diagnosis indicated a profile characterized by marked sensory sensitivity in 10 patients; in three patients, sensory sensitivity was associated with low appetite; and in two cases, low sensory sensitivity was combined with a fear of aversive consequences. In three patients, the clinical diagnosis was not accompanied by a description of the prevalent profile. Two of the patients diagnosed with ARFID had comorbid ADHD, while one had generalized anxiety disorder.

For all participants, cognitive functioning was assessed using four subscales (i.e., similarities, vocabulary, matrix reasoning, and block design) from the Wechsler Intelligence Scale for Children, Fifth Edition (Wechsler 2014) (WISC-V, ages 12 to 16), or the Wechsler Adult Intelligence Scale, Fourth Edition (Wechsler 2008) (WAIS-IV, ages 16 to 20). Anxiety level was measured using State–Trait Anxiety Inventory according to their age (STAI; adult version ((ages 16 through 20) (Spielberger et al. 1983), or child version ((ages 12 through 16) (Spielberger 1973). Finally, information about participants’ height, weight, and handedness, was also recorded.

This study was approved by the local Ethics Committee (CCER, Project-ID 2023 − 01718) and was performed in accordance with the Declaration of Helsinki. Participants aged 16 and older, as well as the parents of those under 18, provided written informed consent. All participants received a gift voucher as compensation for their participation.

### EEG Data Acquisition

EEG data was recorded using 256-channel Geodesic Sensor Net (Electrical Geodesic Inc., OR, USA), at a sampling rate of 1000 Hz and using the vertex (Cz electrode) as a reference. Electrode impedances were kept below 50 KOhms. During EEG recording, participants were seated in a comfortable chair, using a chin rest to reduce head-movement artifacts. Resting-state data were recorded for 5 min per participant after an experimental task engaging executive memory. Participants were instructed to keep their eyes closed, and to remain awake and as calm as possible during the session. The experiment was conducted in an optically, acoustically, and electrically shielded room.

### EEG Preprocessing

The data were processed using a second-order Butterworth band-pass filter (1–40 Hz) along with a 50 Hz notch filter. EEG data were reduced to 204 channels to eliminate muscle artifacts from the neck and face (Berchio et al. 2019; Berchio, Rodrigues, Berchio et al. 2019a, b). Muscle movement artifacts were visually inspected and the contaminated epochs were labeled as artifacts. Approximately 1 min of artifact-contaminated data (see table S2) was manually rejected. Subsequent pre-processing and analyses were performed on ~ 4 min of clean data. Independent component analysis was then applied to correct for electrocardiogram and oculomotor artifacts. Bad channels, if any, were corrected using 3D spline interpolation. All noise-removal summary statistics and additional sanity checks are provided in the supplementary file. Finally, the data were re-referenced to a common average and down-sampled to 250 Hz before further analysis (see following section on *Microstate Analysis*). All EEG preprocessing was done using MATLAB 2018b and Cartool Software v5.01 (Brunet et al. 2011).

### Microstate Analysis

Microstate analysis was performed to identify the optimal number of maps that effectively represent the EEG dataset (Michel and Koenig 2018). A two-step k-means clustering method (Pascual-Marqui et al. 1995) was applied. First, clustering was performed at the individual level to create representative maps for each subject. Second, clustering was conducted at the group level to produce a single set of maps representative of the entire dataset (Bochet et al. 2021; Damborská et al. 2019). At the individual level, the cluster range was set from 1 to 12, and the number of repetitions for the random map initialization process was set to 300 (temporal window half-size = 3 time frames; strength = 10). The same parameters were used for the group-level clustering, with the additional constraint of merging maps that were correlated at 80% or higher. The clustering was performed at time points corresponding to the local maxima of the global field power (GFP) to enhance the signal-to-noise ratio (Murray et al. 2008). The optimal number of maps at all clustering levels was determined using the ‘meta-criterion’ available in Cartool, which identifies the best number of clusters by integrating multiple metrics (Michel and Koenig 2018).

The representative maps identified at the group level were back-fitted to the preprocessed EEG data to extract microstate variables for statistical analysis. At each time point, the spatial EEG pattern was correlated with the representative maps, assigning the map with the highest correlation (Brunet et al. 2011). The spatial correlation threshold was set at 0.5. The fitting process disregarded polarity, and a temporal smoothing factor was applied (temporal window half size = 3 time frames, strength = 10) to minimize noise during low GFP phases (Brunet et al. 2011).

Following the fitting procedure for each microstate, four parameters were extracted: (1) global explained variance (GEV), (2) mean duration, (3) time coverage, and (4) occurrence. The GEV is calculated by summing the explained variances, weighted by the mean of the GFP at each time point, and then converting this into a percentage. The mean duration quantifies the average time (in milliseconds) that a specific microstate remains stable before transitioning to another. The time coverage is the percentage of total time spent in a given microstate. Occurrence indicates the number of microstates of a given class per second. These variables were extracted to investigate group differences in microstate characteristics. Microstate parameters were compared using statistical tests appropriate for the data distribution. To account for multiple comparisons and the intercorrelation among microstate variables, we followed the procedure proposed by Li and Ji (2005), which estimates the effective number of independent tests (M) and applies the *Sidak* correction accordingly. Then, the null hypothesis was rejected for any test with p-values greater than 0.009 (see also Mauriello et al. 2022).

Furthermore, a topographical analysis of variance (TANOVA) was conducted to investigate the similarities between the topographies of the microstate maps in the ARFID and HC groups (for technical details, see Koenig and Melie-García (2010)**)**. Permutations were performed 5,000 times, with a significance threshold set at α = 0.05.

### Transitional Probability

To analyze microstate transition probabilities between groups, we employed Markov chains, calculating transition probabilities for each microstate map to all other maps. Observed probabilities were normalized by dividing them by expected probabilities to account for variability in map occurrence (Bochet et al. 2021; Lehmann et al. 2005). Unpaired t-tests, were used to compare transition probabilities between ARFID and HC groups. The same procedure described above was applied to control for multiple comparisons among microstate transition variables, accounting for their intercorrelation. Specifically, we re-estimated the effective number of independent tests (M) following the method proposed by Li and Ji (2005), and applied the *Sidak* correction accordingly. Based on this, the null hypothesis was rejected for any test with p-values greater than 0.008.

### EEG Source Imaging

On the set of maps where significant group effects were identified, between-group differences in brain sources were investigated using a linear distributed inverse solution model (i.e., standardized low-resolution brain electromagnetic tomography, sLORETA; Pascual-Marqui 2002). Inverse solutions were computed using a 4-shell Locally Spherical Model with Anatomical Constraints (LSMAC; Michel and Brunet 2019), based on 4,000 grey matter voxels from an average pediatric brain template (McGill Centre for Integrative Neuroscience [MCIN], 10–14 years old, https://nist.mni.mcgill.ca/atlases/, Fonov et al. 2011). The data were normalized using a z-score transformation implemented in Cartool (for technical details, see Michel and Brunet 2019). Individual current densities were then averaged across all time points assigned to each microstate. sLORETA source estimations were computed and compared between groups. To account for multiple comparisons, brain network analyses were performed using a randomization test implemented in Cartool, based on 94 regions of interest (ROIs) from the Automated Anatomical Atlas (Tzourio-Mazoyer et al. 2002), with a significance threshold of *p* < 0.05. Post hoc analyses were conducted using two-tailed Student’s t-tests.

### Frequency Analysis

Finally, to exclude larger biases related to activity in specific frequency bands, a power analysis was conducted on the overall EEG data. EEG signal from both groups were decomposed into several frequency bands of interest (namely delta: 0.5–4 Hz, theta: 4–8 Hz, alpha: 8–14 Hz, low beta: 14–26 Hz, high beta: 26–40 Hz). The frequency range for different bands were determined using a combination of data-driven approaches alongside canonical EEG band definitions. We first computed grand average power spectra separately for both groups and then averaged them to create a combined spectrum representing the full sample. This combined spectrum was then analyzed using k-means clustering on the frequency and power data, revealing natural groupings that guided our definitions of low beta (14–26 Hz) and high beta (26–40 Hz). For delta, theta, and alpha bands, we referred to established canonical ranges from the literature (e.g., Newson and Thiagarajan 2019), ensuring our band definitions are both empirically grounded and aligned with prior studies. The power of each frequency was calculated using the multi-taper method with a Hanning taper (method = ‘mtmfft’) and a frequency resolution of 0.034 Hz across the entire time window of the 5-minute resting-state data. Power was extracted for each frequency band of interest and was averaged across all channels and time windows in the ARFID and HC groups separately. This analysis was performed in MATLAB 2018b. Group differences were calculated using statistical analyses appropriate to data distributions.

### Correlations of Microstate Parameters with Overall Functioning

To investigate the potential links between microstate dynamics and symptom severity, either Spearman’s rank correlations or Pearson’s correlation (confidence interval determined using standard bootstrapping method with 5000 randomizations), were employed to assess the relationship between the four WISC subscale scores (similarities, vocabulary, matrix reasoning, and block design), STAI-state and STAI-trait measures, and BMI with the three extracted microstate parameters mean duration, time coverage, and occurrence. To control for multiple testing and reduce the likelihood of Type-I errors, the false discovery rate (FDR) correction was applied to all statistical analyses.

To explore additional links between microstate transitions and the cognitive and clinical factors mentioned earlier, we conducted further correlations using the same statistical method. All statistical analyses were performed using R software (v4.4.1).

## Results

### Demographic and Clinical Variables

ARFID patients and HC participants had a similar age (t_34_ = 1.65, *p* = 0.108, d_cohen_= 0.55). ARFID patients, on the other hand, had a significantly lower BMI compared to HC (t_34_ = 3.04, *p* = 0.005, d_cohen_= 1.01). All participants included in the study were right-handed.

Further, statistical significance was observed in the STAI; state anxiety levels were significantly higher in ARFID patients compared to the HC group, while trait anxiety showed only marginal significance (STAI State: U = 46, *p* = 0.00024, δ = −0.72; STAI Trait: U = 100, *p* = 0.051, δ = −0.38) (see Fig. S1).

No significant group differences were found for any of the four subscales of the intelligence scale (i.e., block design, similarities, matrix reasoning, and vocabulary) (Vocabulary: U = 126, *p* = 0.265, δ= −0.22; Matrix reasoning: U = 158.5, *p* = 0.923, δ= −0.022; Similarities: t_34_ = 0.88, *p* = 0.388, d_cohen_= 0.29; Block design: U = 111, *p* = 0.108, δ= −0.315).

The summary of all the demographic and clinical information is given in Table 1.

**Table 1:** Demographic and clinical data of the study sample

	*HC (n = 18)*		*ARFID (n = 18)*		
	Mean	SD	Mean	SD	*p*-value
**Age**	14.56	2.85	12.78	3.57	0.108
Height*	1.63	0.09	1.52	0.15	0.011
Weight*	52.98	10.88	41.03	10.49	0.002
BMI*	19.78	2.88	17.27	1.996	0.005
**STAI**					
*Trait*	33	6.54	38.75	9.74	0.051
State*	27.89	4.79	40.38	9.83	0.00024
**WISC**					
*Block Design*	10.22	3.26	11.72	2.67	0.141
*Similarities*	13.11	3.05	12.22	2.96	0.381
*Matrix reasoning*	10.33	2.70	10.77	2.58	0.617
*Vocabulary*	12.39	3.09	13.27	3.23	0.405

Statistically significant differences between groups are indicated with an asterisk (*p* < 0.05)

### Microstate Results

**Fig. 1:**
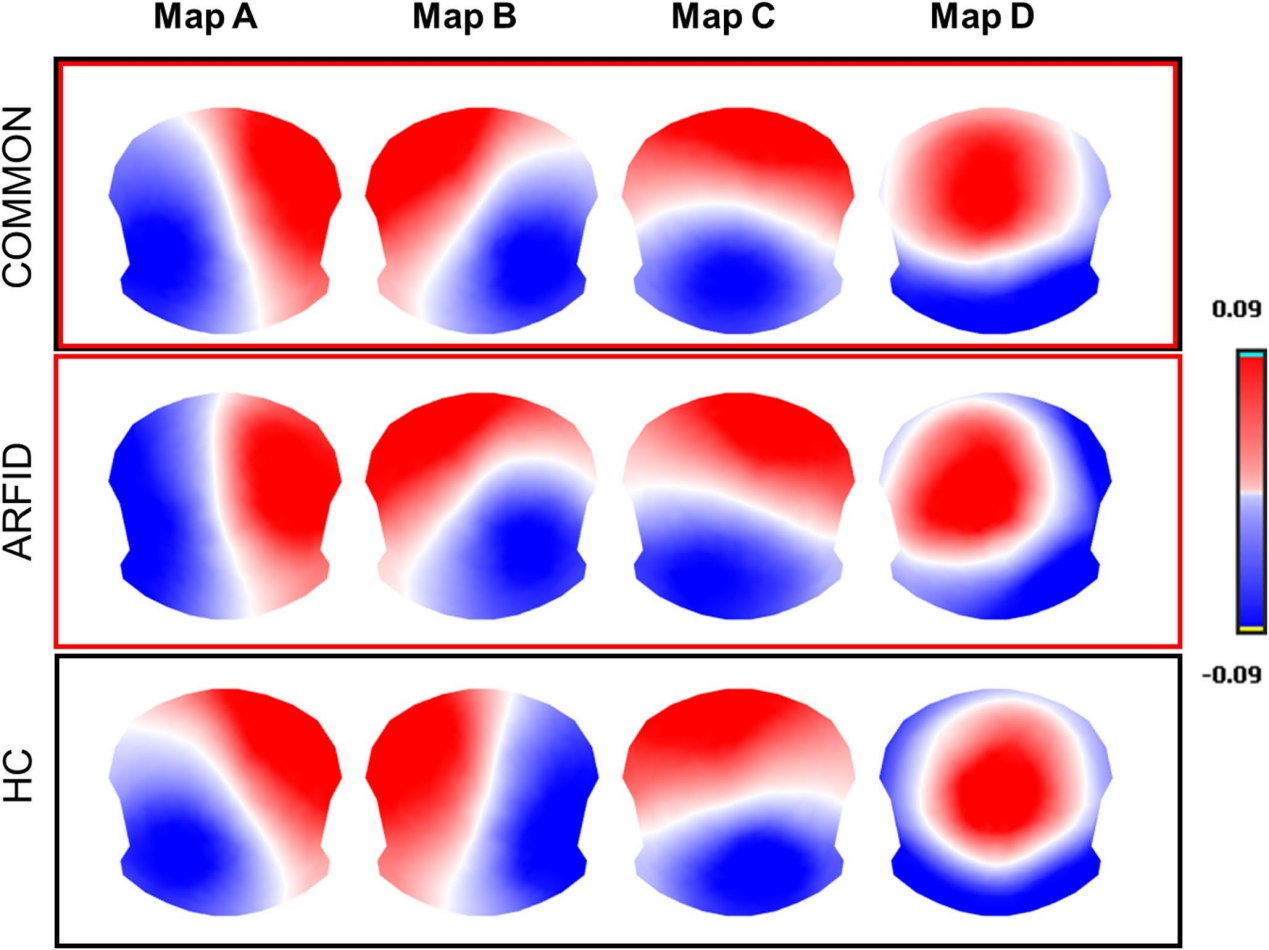
Microstate scalp maps. Four microstates were identified across all participants (COMMON) and within the ARFID, and HC groups

The meta-criterion identified the four most prominent maps across all participants, during second level clustering (both groups together), collectively explaining 76.32% of the global explained variance (GEV) in the entire EEG dataset. The topographies of these maps were similar to those previously described as maps A, B, C, and D (Custo et al. 2017; Khanna et al. 2015; Koenig et al. 2002; Michel and Koenig 2018). Based on prior literature, the maps were labeled from A to D, with map A showing a left posterior-right anterior orientation, map B a right posterior-left anterior orientation, map C an anterior-posterior orientation, and map D having a centro-frontal maximum (Fig. 1). Additionally, we examined the prototypical maps that best characterized the datasets for both ARFID patients and HC separately. The meta-criterion during this second level clustering (but for each group separately) revealed four resting-state maps in both ARFID and HC groups, explaining 76.49% and 75.83% of the global variance, respectively. To confirm that these microstate classes were consistent across groups, Pearson’s spatial correlations were performed for each class. The analysis showed moderate similarity between the groups’ maps, with Pearson correlation coefficients of 0.81 (A-A), 0.81 (B-B), 0.86 (C-C), and 0.77 (D-D). The TANOVA comparison between the two groups showed no significant statistical difference (*p* > 0.05). As a result, the four cluster maps identified across both groups were retained for further analysis.

**Fig. 2:**
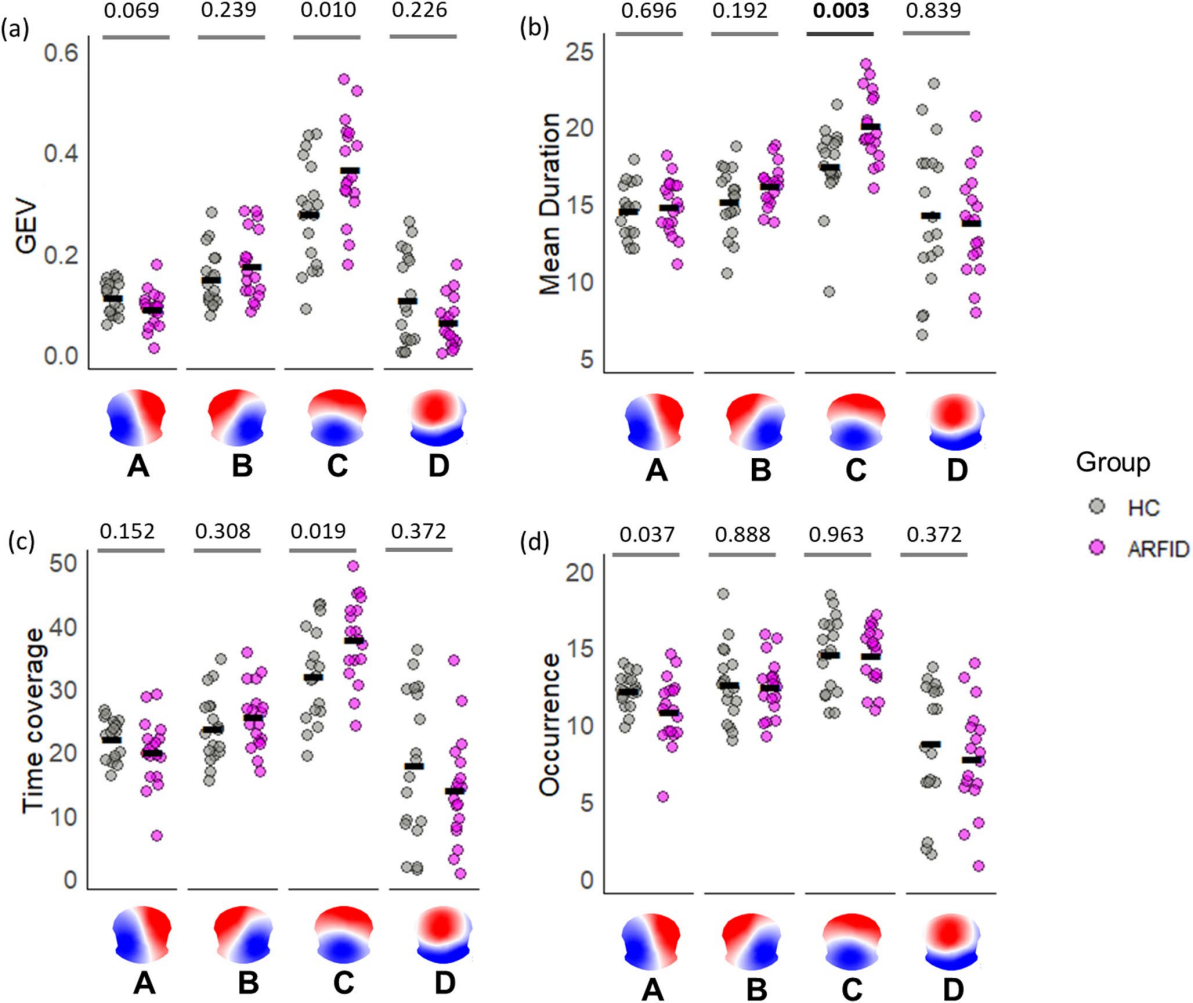
Microstate parameters analysis. Characteristics of the four microstates extracted using a single set of maps representative of the whole dataset: Global explained variance (GEV) (**a**), mean duration (**b**), time coverage (**c**), and occurrence (**d**). Individual values are plotted in gray for HC and pink for ARFID patients. The significant difference that survived multiple comparison is indicated by p-values in bold font

Since some microstate parameters were not normally distributed, Mann-Whitney U tests were conducted to investigate group differences (ARFID-HC) in microstate features. Significant differences between groups, after correction for multiple comparisons, were found only for the mean duration of Map C (U = 254, *p* = 0.003, δ = 0.57), with the ARFID group showing higher values than the HC group (Fig. 2). No other maps or parameters showed statistically significant differences that survived multiple comparison correction (all corrected *p* > 0.009).

### Transition Probability

**Fig. 3:**
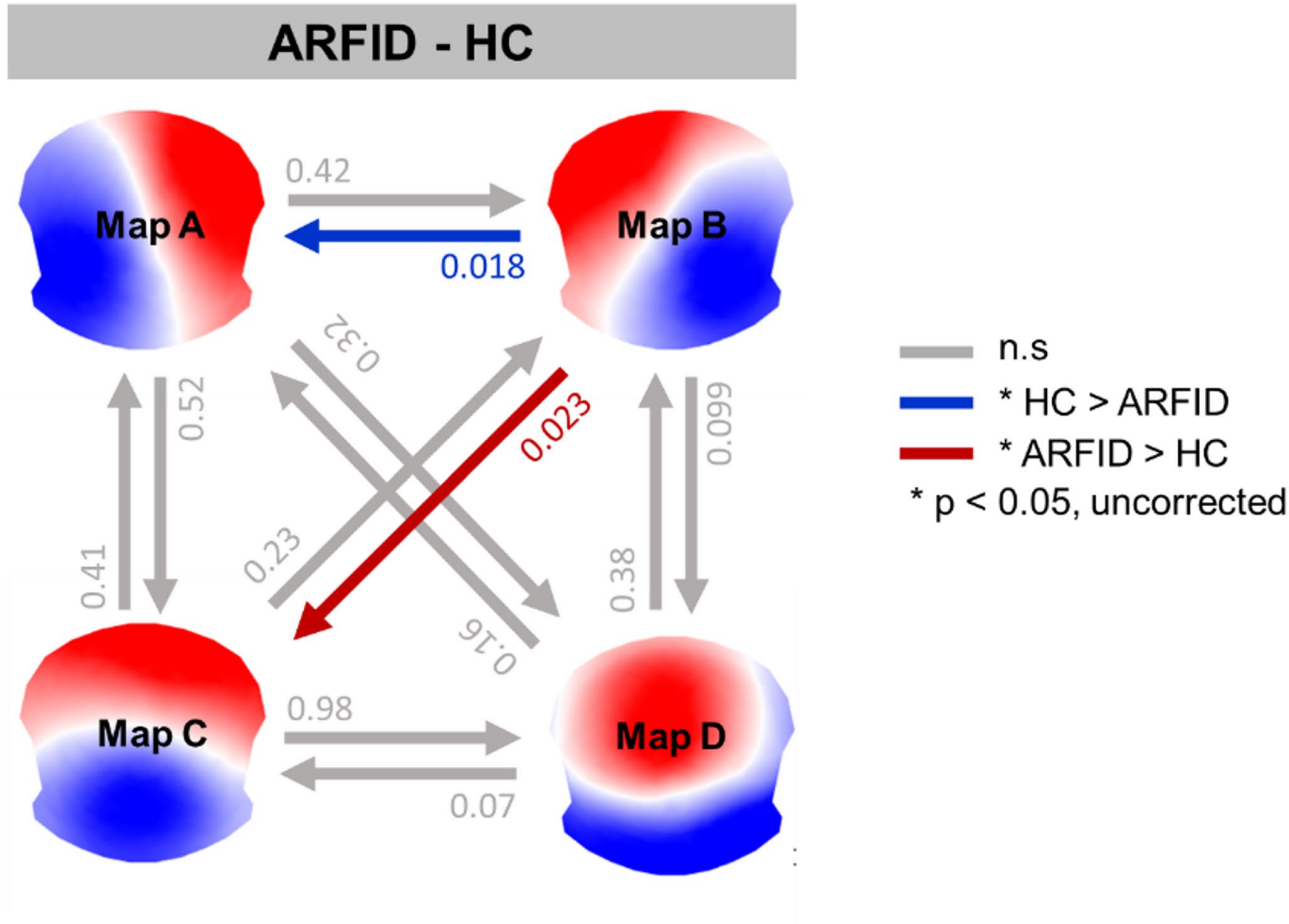
EEG microstate transition dynamics using Markov chains. Comparison of the transition probabilities between ARFID and HC. Colored arrows indicate statistical trends (*p* < 0.05); grey arrows represent effects with p-values greater than or equal to 0.05. Blue arrow: transition probabilities in HC>ARFID; red arrow: transition probabilities in ARFID>HC

Based on the four maps, 12 possible pairs of transitions were investigated between ARFID and HC (see, Table 2). No group differences in transition probabilities remained significant after *Sidak* correction (all corrected *p* > 0.008). Nevertheless, the uncorrected p-values indicated decreased transition probabilities from microstate B to A in the ARFID group compared to HC (*p* = 0.018; Cohen’s d = 0.82), while transition probabilities from microstate B to C were increased in ARFID compared to HC (*p* = 0.023; Cohen’s d = 0.80) (see Fig. 3). Although the t-test contrasts were only significant at the uncorrected level, we report them here as trends worth considering, particularly given the small sample size, and as possible indicators of differential patterns in microstate parameters.

**Table 2:** Summary of transition probability

	HC (*n* = 18)		ARFID (*n* = 18)		
Transition	Mean	SD	Mean	SD	*p*-value
A to B	1.03	0.05	1.04	0.07	0.422
A to C	1.09	0.05	1.10	0.04	0.525
A to D	0.82	0.16	0.76	0.16	0.320
B to A*	1.02	0.06	0.97	0.06	0.019
B to C*	1.06	0.09	1.13	0.07	0.023
B to D	0.898	0.11	0.83	0.13	0.099
C to A	1.06	0.07	1.04	0.08	0.410
C to B	1.02	0.06	1.05	0.09	0.233
C to D	0.94	0.14	0.94	0.11	0.983
D to A	0.86	0.10	0.80	0.12	0.164
D to B	0.97	0.05	0.95	0.08	0.378
D to C	1.07	0.099	1.12	0.07	0.071

* *p* < 0.05, uncorrected

### Between-group Source Imaging Analyses

**Fig. 4:**
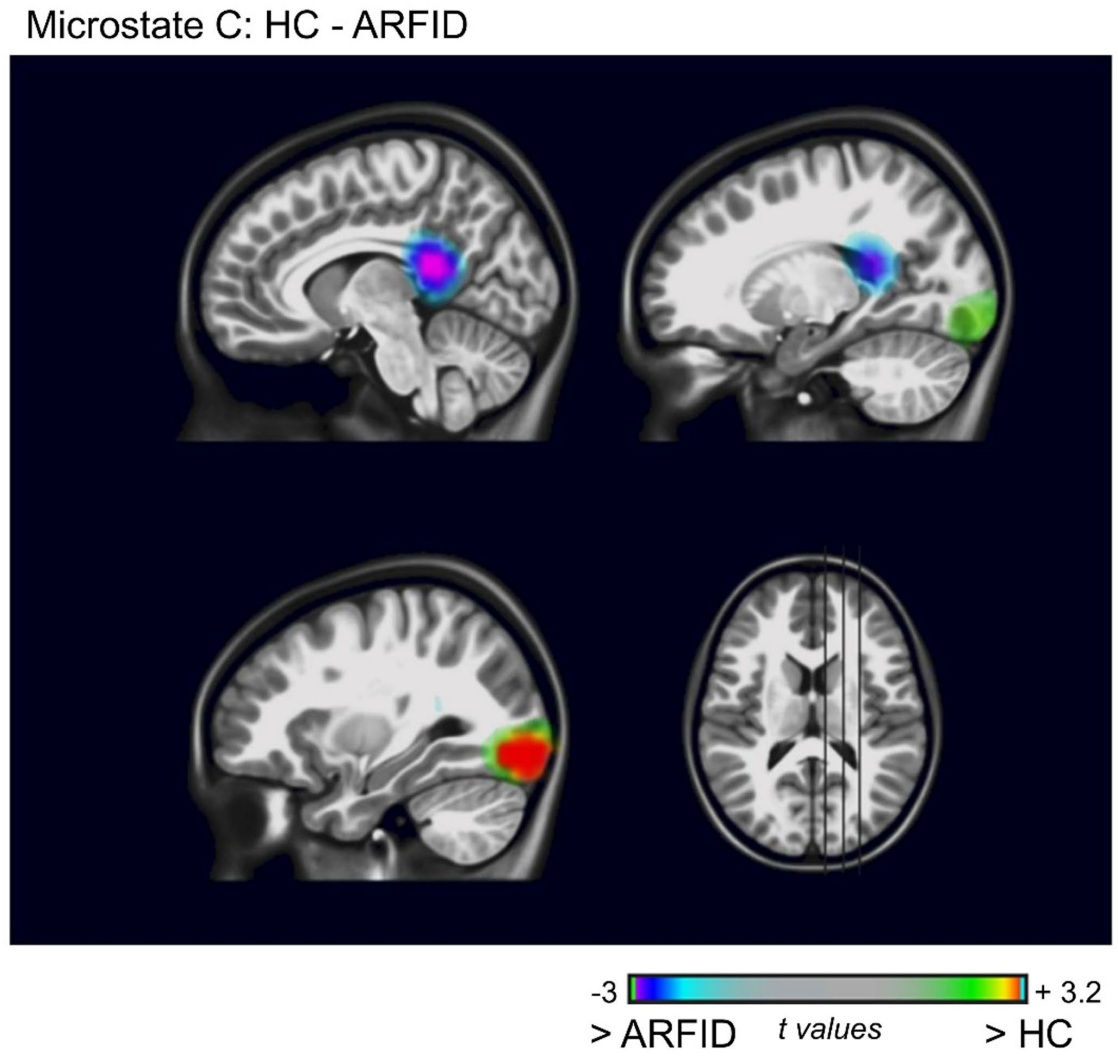
Source differences for microstate C between the avoidant/restrictive food intake disorder (ARFID) group and healthy controls (HC). The color bar represents the t-values (cluster level, *p* < 0.05): purple to light-blue colors indicate increased activation in ARFID, and red to green-values indicate increased activation in HC

Source imaging analyses were conducted for microstate C, which showed significant differences between the two groups. For microstate C, the randomization test revealed differences between the ARFID and HC groups in the right posterior cingulate cortex and the right inferior occipital cortex (all p’s < 0.05). Increased activation was observed in patients with ARFID compared to HC in the right posterior cingulate cortex (t = −3.012, *p* = 0.005, Cohen’s d = 1.004). In contrast, decreased activation was found in the right inferior occipital cortex (t = 3.223, *p* = 0 0.003, Cohen’s d = 1.074) (Fig. 4).

### Cognitive and Clinical Correlations

**Fig. 5:**
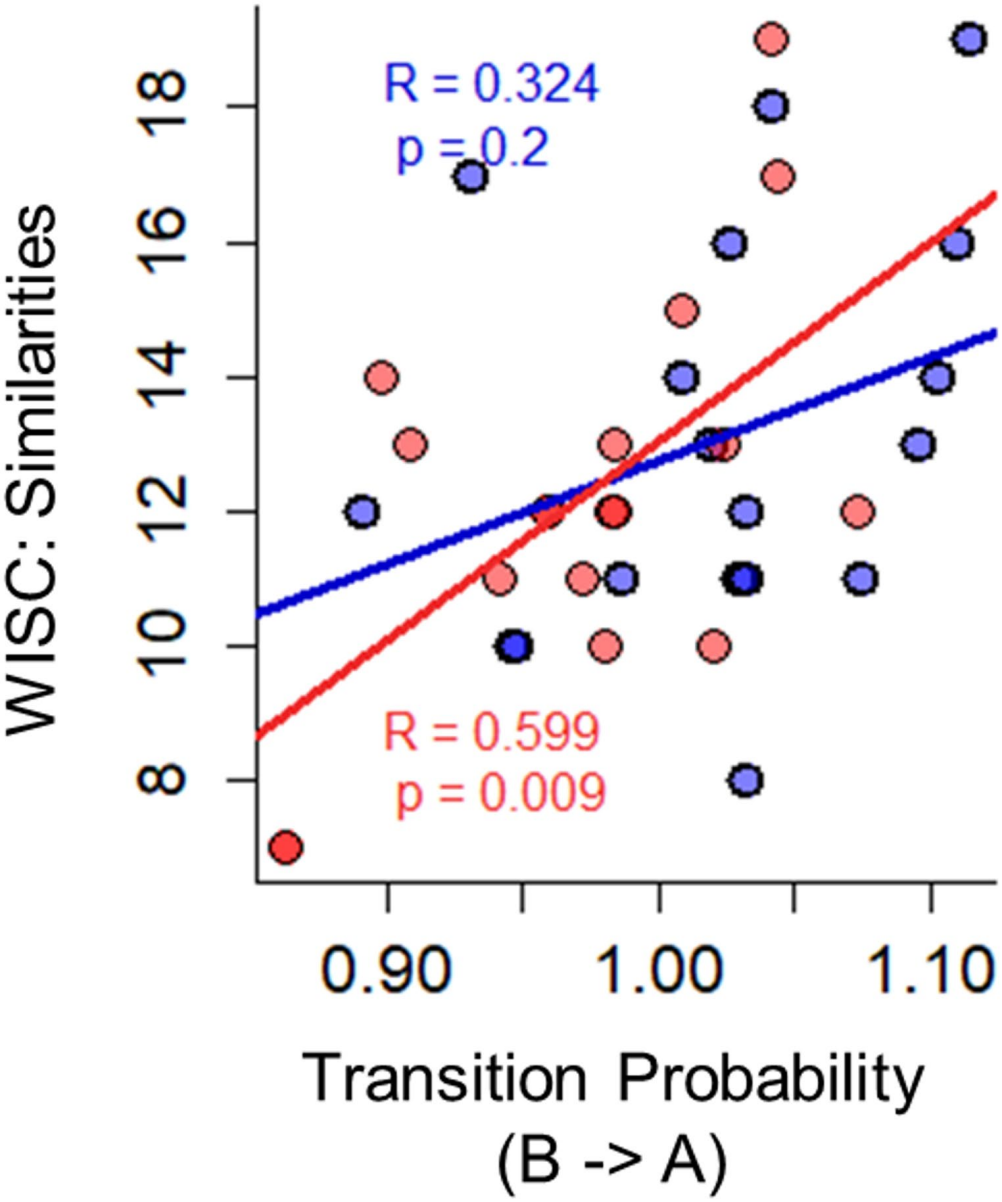
Correlations with WISC subscale Similarities: Significant correlation was found between cognitive parameter WISC subscale *Similarities* with transition from Map B → A in ARFID (represented in red), but not in HC (represented in blue)

Correlation analyses were conducted separately in the ARFID and HC groups to examine the relationship between the GEV, mean duration of microstate C, and transitional probabilities (B → A and B → C) with other clinical and cognitive parameters. We found in the ARFID group, but not in HC group, a significant positive association between WISC subscale Similarities and the transition from B → A (Pearson’s *r* = 0.599, *p* = 0.009, CI = 0.10, 0.87) (see Fig. 5). No other effects were found to be significant or survived significance after FDR correction either in the ARFID or in HC group in any other parameters (all p’s > 0.05).

### Frequency Analysis

**Fig. 6:**
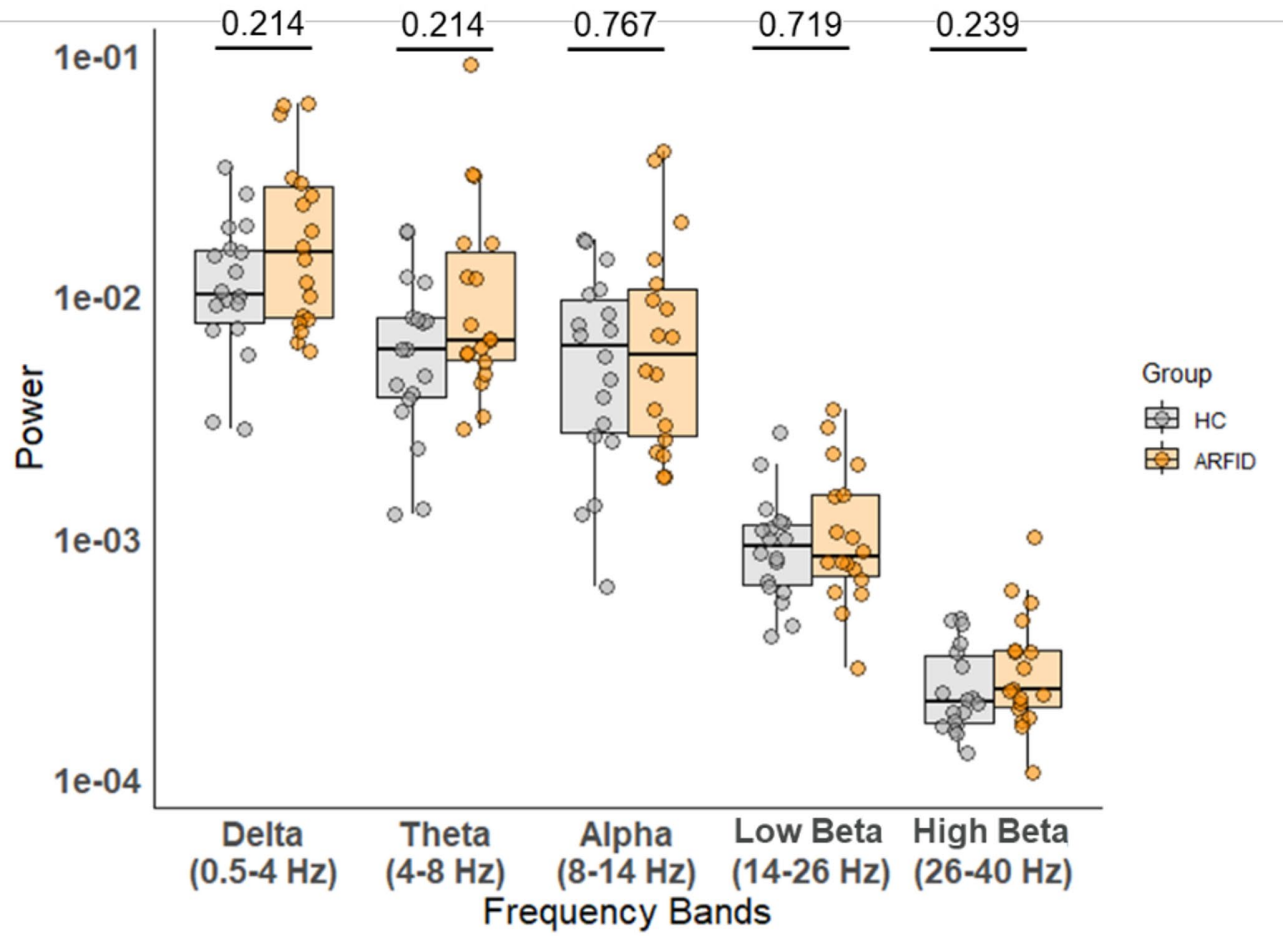
Frequency Analysis: No significant group differences were found across 5 different frequency bands- delta (0.5–4 Hz), theta (4–8 Hz), alpha (8–14 Hz), low beta (14–26 Hz), and high beta (26–40 Hz)

In the frequency analysis, a Mann-Whitney U test revealed no statistically significant differences in spectral power between the ARFID and HC groups in any of the frequency bands (delta: U = 202, *p* = 0.214; theta: U = 202, *p* = 0.214; alpha: U = 172, *p* = 0.767; low beta: U = 175, *p* = 0.596; and high beta: U = 200, *p* = 0.239) (see Fig. 6). This analysis therefore excluded major bias in the overall basic EEG activity between the two groups.

## Discussion

This study aimed to provide evidence of resting-state EEG microstate dynamics in children and adolescents with ARFID. To our knowledge, this is the first study to investigate neurophysiological alterations in ARFID using microstate analysis, offering a novel perspective on brain network abnormalities in this population. Our findings indicate preliminary differences in EEG microstate parameters in children and adolescents with ARFID, particularly in microstate C mean duration. These results suggest potential differences in resting-state brain dynamics that warrant further investigation. These results contribute to the growing body of research indicating that ARFID is not solely a behavioral disorder but may also have a neurobiological basis, potentially linked to altered brain network dynamics.

### Microstate C in ARFID

One of the most notable findings of this study was the significant increase in the mean duration of microstate C in the ARFID group compared to the HC group. Microstate C has previously been associated with both the salience network and the DMN (Britz et al. 2010; Hu et al. 2021; Nishida et al. 2013). The salience network plays a critical role in detecting and responding to personally relevant environmental stimuli, including interoceptive signals such as hunger and satiety (García-García et al. 2012; Sewaybricker et al. 2019; Wijngaarden et al. 2015), while the DMN is involved in internally directed thought and self-referential processes (Menon 2023). However, microstate C is the most difficult to consistently identify, as its topographical patterns show some variability across studies (Tarailis et al. 2024). To support the interpretation of microstate C in our data, we conducted source imaging analyses. These analyses revealed increased activation in the right posterior cingulate cortex, a region commonly associated with the DMN (Davey et al. 2016; Uddin et al. 2009), in ARFID compared to HC. Conversely, we observed decreased activation in the right inferior occipital cortex in ARFID. These results may point to a potential dysregulation of a key node of the DMN, possibly reflecting increased engagement in internally focused processes and reduced involvement in external sensory processing in the ARFID group. Previous studies have implicated the role of the posterior DMN in food intake-related behavior (Cerit et al. 2019; Tregellas et al. 2011). This imbalance between internal and external control, potentially present even at rest, could lead some individuals with ARFID to avoid food in order to prevent experiencing certain internal sensations (Zucker et al. 2019).

Alternatively, it could be hypothesized that the increased engagement of microstate C is related to anxiety features in ARFID, considering the association between the DMN and anxiety reported in previous studies (Coutinho et al. 2016; Zhao et al. 2007). ARFID patients often avoid food due to fears of negative consequences, such as choking or vomiting (Brigham et al. 2018; Fonseca et al. 2024). This heightened anticipatory anxiety might contribute to sustained activation of internally oriented networks, such as the DMN, potentially reflecting a persistent focus on internal threat-related thoughts or sensations. However, our results do not support the hypothesis that increased engagement of microstate C is directly related to anxiety traits or state, as we did not find significant correlations between microstate C parameters and anxiety levels in ARFID patients. Therefore, the prolonged duration of microstate C observed in ARFID patients may instead reflect a failure to deactivate this brain network rather than being a direct effect of anxiety.

Nonetheless, we acknowledge that interpretations linking microstates to specific resting-state networks remain somewhat speculative, and should be considered as hypotheses rather than definitive conclusions. Further multimodal imaging studies will be needed to clarify these associations.

### Transition Dynamics and Network Dysregulation

Although no effects survived correction for multiple comparisons, our analysis of transition probabilities, used to quantify the likelihood of switching from one microstate to another, revealed trends suggesting potential differences between ARFID and HC. Specifically, this trend highlighted potential differences in the transitions from microstate B → A and microstate B → C. The trend toward reduced transition probability from microstate B → A in ARFID patients may indicate differences in the visual-auditory networks typically associated with microstates A and B (Britz et al. 2010; Tarailis et al. 2024). Patients with ARFID often display heightened sensitivity to the sensory properties of food (e.g., taste, texture) (Brigham et al. 2018; Fonseca et al. 2024; Zimmerman and Fisher 2017). Such a pattern, although preliminary, might reflect broader difficulties in integrating multisensory information, which could underlie the sensory-related avoidance behaviors observed in ARFID (Brigham et al. 2018; Thomas et al. 2017; Willmott et al. 2024; Zimmerman and Fisher 2017). Conversely, the trend toward increased transition probability from microstate B → C in ARFID patients may suggest a different interaction between the visual network (microstate B) and the DMN (microstate C). This heightened interaction could indicate that visual cues trigger a more intense DMN response in ARFID patients compared to HC. Such a mechanism, if confirmed, might contribute to the amplification of aversive reactions to food-related stimuli and intensify the lack of interest in eating observed in some individuals with ARFID.

Notably, the reliability of microstate transition metrics, particularly those based on first-order Markov chain models, has been shown to be limited across studies (Antonova et al. 2022; Bagdasarov et al. 2024; Kleinert et al. 2024). Given the small sample size and the exploratory nature of the current analyses, interpretations should, therefore, remain cautious, and further work is needed to determine the replicability and functional relevance of these transition trends.

The findings of this study also highlighted a significant correlation between the transition probability from microstate B to A and the WISC Similarities subscale in the ARFID group but not in HC. Specifically, a higher probability of transitioning from B to A was associated with higher WISC Similarity scores. This preliminary finding may point to a possible link between sensory brain network differences in ARFID and cognitive processing, particularly in verbal concept formation, reasoning, and abstract thinking. Hypothetically, such alterations in brain dynamics could reflect broader cognitive characteristics of ARFID (Basile et al. 2021; Mahr et al. 2023), potentially impacting daily functioning in this population. However, these results should be interpreted with caution, as participants in this study were not engaged in a cognitive task, and no experimental manipulations were used to directly assess the cognitive relevance of these microstate transitions.

### Implications for Neurobiological Models of ARFID

Our findings provide important insights into the neurobiological underpinnings of ARFID concerning the three-dimensional model of ARFID neurobiology (Thomas et al. 2017), particularly in relation to the distinct brain network dynamics at rest. The increased duration of microstate C in ARFID patients may reflect overactivation of the DMN, potentially indicating heightened self-referential processing (Hu et al. 2021; Nishida et al. 2013). The concurrent increase in activity in the posterior cingulate cortex and decrease in visual regions further suggest that ARFID may involve differential interactions between internal control mechanisms and sensory processing networks. Such an imbalance could contribute to the reduced interest in food observed in these individuals.

In this study, in accordance with the model proposed by Thomas et al. (2017), we hypothesized that hyperactivation of the defense motive system might manifest in children and adolescents with ARFID as differences in microstate D. However, this hypothesis was not supported by our findings. This suggests that psychophysiological reactivity to fear stimuli may only become evident under specific circumstances, such as during the processing of anxiety-provoking stimuli. This hypothesis aligns with previous research that has demonstrated hyperactivation in emotional brain regions, such as the orbitofrontal cortex and insula, specifically during food anticipation in patients with ARFID (Kerem et al. 2022). Consequently, capturing these dynamics during resting-state measurements of brain networks may be more challenging, as the activation of these systems may not be as pronounced in a baseline state. Nevertheless, it is important to note that most of the ARFID patients included in this study predominantly exhibited a profile characterized by heightened sensory sensitivity. Overall, our findings appear consistent with the model proposed by Thomas et al. (2017) and further suggest that microstate power analyses could be a useful tool for identifying specific dimensions associated with ARFID clinical profiles.

Furthermore, the neurobiological alterations observed in ARFID also raise important questions about the potential for targeted interventions. Current treatments for ARFID primarily rely on behavioral therapies such as cognitive-behavioral therapy (CBT), family-based therapy and exposure therapy (Howard et al. 2023; Thomas et al. 2018), but the identification of specific neural markers could open the door to more tailored interventions. For example, neurofeedback training aimed at modulating the brain activity (Bhadra et al. 2025; Hamilton et al. 2016; Ros et al. 2013) related to the DMN or improving sensory integration may hold promise for reducing avoidance behaviors in ARFID patients and food-related anxiety. However, further research is needed to explore these possibilities.

### EEG Microstates in Eating Disorders

Studies investigating the dynamics of EEG microstates in ED are rather limited. To our knowledge, only a single study has examined these dynamics during resting-state in adolescents with AN (Berchio et al. 2024). This study highlighted that adolescents with AN exhibit a reduced occurrence and time coverage of microstate C compared to the control group. This finding suggests that the differences associated with microstate C could constitute a characteristic related to ED. However, the reduced presence of microstate C in AN, in contrast to its greater manifestation in ARFID, may indicate a distinctive neural trait between the two disorders.

This preliminary evidence suggests that microstate analyses can effectively identify specific markers associated with different ED. Contrary to findings in anorexia nervosa, where an increased B→A transition probability has been reported (Berchio et al. 2024), the present ARFID sample showed a decreased probability of transitions from microstate B to A (trend-level). This divergence suggests that microstate dynamics may differentiate eating-disorder subtypes rather than reflect a uniform alteration across ED. Even so, these preliminary data support the utility of EEG microstate analyses for identifying disorder-specific markers, while possible transdiagnostic effects remain to be clarified in larger samples.

### Limitations and Future Directions

While this study provides valuable insights into the neurophysiology of ARFID, several limitations must be acknowledged. This study is exploratory in nature, and the findings should be interpreted with caution. The small sample size of ARFID patients (n = 18) and the large number of statistical comparisons limit the generalizability of our findings. Although we applied corrections for multiple comparisons, such as independent contrast adjustments or FDR corrections across different analyses, some observed effects (e.g., transition probabilities) did not survive correction. Consequently, the sample size may have limited sensitivity to detect smaller effects and may have prevented us from investigating subgroup-specific effects, such as those related to different ARFID clinical profiles. Additionally, we acknowledge as a limitation that the relatively high % change in variance following ICA may reflect substantial artifact contamination and, in some cases, poor signal-to-noise ratio. The observed association between the WISC subscale Similarities and the B → A transition in ARFID is correlational and should not be interpreted as evidence of a causal relationship. Moreover, the cross-sectional design precludes conclusions about causal relationships between brain network abnormalities and ARFID symptomatology. Because data were collected at a single time point, the observed neural alterations may primarily reflect the current symptom profile rather than underlying mechanisms contributing to the development of ARFID. Longitudinal studies are needed to determine whether these neural patterns remain stable over time or change with symptom fluctuations. Three participants presented comorbid conditions (e.g., ADHD, generalized anxiety disorder), which may constitute potential confounding factors. Due to the limited sample size, it was not feasible to conduct separate analyses to control for these variables. Nevertheless, the clinical heterogeneity observed in our sample reflects the real-world complexity of the ARFID population and should be considered when interpreting the findings.

While this study focused on resting-state EEG, future research should also investigate task-related EEG dynamics to explore how neural network activity changes in response to food-related stimuli. This could provide further insights into the neural mechanisms underlying food avoidance and sensory sensitivity in ARFID. In addition, integrating EEG findings with other neuroimaging modalities, such as fMRI, could provide a more comprehensive understanding of the large-scale brain networks involved in ARFID. Future studies with larger samples and more comprehensive methods are needed to confirm and expand upon these preliminary findings.

## Conclusion

In conclusion, this study provides preliminary evidence that children and adolescents with ARFID display distinct EEG microstate dynamics, particularly involving a microstate associated with the salience network and its interaction with the sensory network. These findings suggest that ARFID is linked to altered brain network dynamics, potentially underlying the clinical features that characterize this disorder. Moreover, the findings of this study highlighted specific neural traits that may be characteristic of this ED. While further research is needed to confirm these findings and explore their clinical implications, this study represents an important step toward understanding the neurobiological basis of ARFID and highlights the potential for future neurophysiological interventions.

## Supplementary Information

Below is the link to the electronic supplementary material.

Supplementary Material 1

## Data Availability

No datasets were generated or analysed during the current study.
