# Supplementary material for: EEG Resting-state Microstate Dynamics in Children and Adolescents with Avoidant/Restrictive Food Intake Disorder (ARFID)
Source: Brain Topogr. 2025 Sep 30;38(6):73. doi: 10.1007/s10548-025-01149-4 (PMC12479596; doi:10.1007/s10548-025-01149-4)
Supplement: Supplementary file 1 — Supplementary Material 1 [file 10548_2025_1149_MOESM1_ESM.docx]

# Supplementary Materials

**Participants**

The diagnosis of ARFID was made by certified psychiatrists, according to the Diagnostic and Statistical Manual of Mental Disorders, Fifth Edition (DSM-5) guidelines. All patients included in the study were recruited through ALiNEA ("Alimentation et Nutrition chez l’Enfant et l’Adolescent"), a specialized outpatient clinic at the University Hospital of Geneva (HUG) dedicated to the assessment and treatment of eating and feeding disorders in children and adolescents. The diagnosis of ARFID was established at ALiNEA for 15 patients aged 16 or younger. For three participants older than 16, an initial diagnosis had been made at ESCAL ("Espaces de soins pour les troubles du comportement alimentaire"), also at HUG, which focused on older adolescents and young adults. Based on the DSM-5 criteria, 17 patients received a diagnosis of ARFID, while one met subthreshold criteria, presenting significant symptoms that were not severe or frequent enough to fully meet the diagnostic criteria. According to the diagnoses made by psychiatrists, two patients had comorbid ADHD, and one patient had a comorbid anxiety disorder. See Table S1.

Table S1: Individual profile of ARFID patients with comorbidities

| **Patient ID** | **ARFID or under threshold** | **ARFID type** | **Comorbidities** |
| --- | --- | --- | --- |
| **P01** | ARFID | Sensory, phobic | Generalized Anxiety |
| **P02** | ARFID | Not Established | None |
| **P03** | ARFID | Sensory, low appetite | None |
| **P04** | ARFID | Not established | None |
| **P05** | ARFID | Not established | None |
| **P06** | ARFID | Sensory | None |
| **P07** | ARFID | Sensory | None |
| **P08** | ARFID | Sensory | None |
| **P09** | ARFID | Sensory | None |
| **P10** | ARFID | Sensory | ADHD |
| **P11** | ARFID | Sensory | None |
| **P12** | ARFID | Sensory, low appetite | None |
| **P13** | ARFID | Sensory, low appetite | None |
| **P14** | ARFID under threshold | Sensory | None |
| **P15** | ARFID | Sensory | None |
| **P16** | ARFID | Sensory | None |
| **P17** | ARFID | Sensory, phobic | ADHD |
| **P18** | ARFID | Sensory | None |

P: patient; ID: identifier;

ARFID: Avoidant/Restrictive Food Intake Disorder;

ADHD: Attention-Deficit/Hyperactivity Disorder.

**Data Pre-processing**

The pre-processing statistics are summarized in Table S2.

Table S2: Summary statistics of noise removal during EEG preprocessing across HC and ARFID group

| **Pre-processing metric** | **HC** | | **ARFID** | | ***p-value*** |
| --- | --- | --- | --- | --- | --- |
|  | ***mean*** | ***SD*** | ***mean*** | ***SD*** |  |
| **Duration removed (manual rejection) (sec)** | 68.03 | 44.35 | 60.49 | 11.25 | 0.473 |
| **Bad channels interpolated (count)** | 4.28 | 2.16 | 3.17 | 2.75 | 0.187 |
| **ICA components rejected (count)** | 15.33 | 7.35 | 19.22 | 6.92 | 0.095 |
| **% change in variance** | 28.92 | 50.99 | 21.87 | 81.38 | 0.758 |
| **Signal-to-noise Ratio (after-before)** | 0.61 | 0.59 | 0.48 | 0.52 | 0.485 |

Table S2 presents the summary statistics for noise removal during EEG preprocessing across HC and individuals with ARFID. The average duration of data removed due to manual rejection was slightly higher in HC than ARFID, though this difference was not statistically significant (F(1, 34) = 0.49 ; p = 0.473). Similarly, no significant group differences were observed in the number of bad channels interpolated (F(1, 34) = 1.82 ; p = 0.187), and ICA components rejected (F(1, 34) = 2.66; p = 0.095). To measure the change of variance in the data after ICA cleaning, we calculated ‘% change in variance’ (see equation 1). It reflects the relative change in signal variance following artifact removal and is not bounded between 0–100%, i.e., values can be negative (if variance increases post-ICA) or greater than 100% (if variance approaches zero after artifact removal). Despite the sometimes-large values, ICA components were selected conservatively, and visual inspection confirmed that neural signals of interest were preserved for subsequent microstate analysis. There was no statistical difference between the two groups (F(1, 34) = 0.10 ; p = 0.758).

% change in variance = [1− (Var_after ICA_/ Var_before ICA_)] × 100 …equation 1

Additional sanity check: To further assess data quality, we estimated the signal-to-noise ratio (SNR) based on canonical EEG frequency bands, defining signal as the combined power in theta, alpha, and beta bands, and noise as the combined power in delta and gamma bands (see equation 2). This metric captures the relative preservation of dominant oscillatory activity while accounting for low-frequency drift and high-frequency muscle artifacts. Across all datasets, SNR values were higher after ICA cleaning than before, indicating that artifact removal improved the relative prominence of neural rhythms and there was no statistical difference between the two groups (*F*(1, 34) = 0.50, *p* = 0.485) (Figure S1).

SNR = Power(θ+ɑ+𝛃) / max(Power(δ+γ), ϵ) ...equation 2


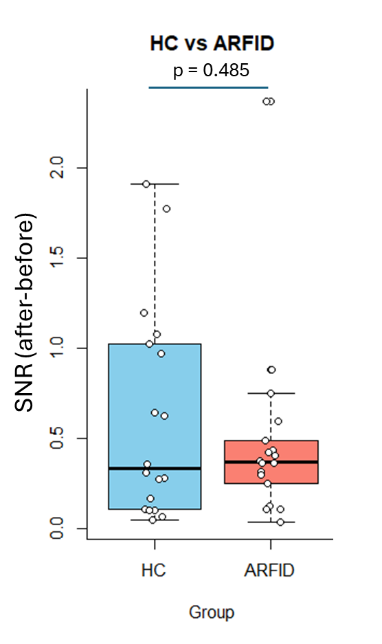


**Figure S1: Group differences for SNR retained after ICA cleaning.**

Overall, the pre-processing metrics were comparable between groups, with none showing statistically significant differences.

**Correlation between the total duration of clean EEG data and microstate temporal parameters**

To address potential concerns regarding variability in the duration of usable EEG data, we examined whether the total length of artifact-free data was systematically related to microstate parameters. Specifically, we computed correlations between the total clean data duration and the mean duration, time coverage, and occurrence of each microstate class (A–D) separately for the ARFID and control groups. Based on the four microstate maps and three temporal parameters, 12 possible pairs of correlation were investigated between ARFID and HC (see, Table S3). No significant associations were observed after applying Sidak correction for multiple comparisons (all corrected *p* > 0.008), indicating that variability in data length did not systematically bias the microstate results.

**Table S3: Correlation analysis between the total duration of clean EEG data and each of the microstate temporal parameters.**

| **Correlation with Data Duration** | **R** | | ***p-value*** | |
| --- | --- | --- | --- | --- |
|  | **HC** | **ARFID** | **HC** | **ARFID** |
| **Occurence–A** | 0.18 | 0.02 | 0.467 | 0.947 |
| **Occurrence–B** | -0.46 | 0.29 | 0.056 | 0.239 |
| **Occurrence–C** | -0.34 | 0.33 | 0.172 | 0.178 |
| **Occurrence–D** | 0.28 | -0.27 | 0.261 | 0.287 |
| **Mean Duration–A** | 0.37 | -0.18 | 0.130 | 0.486 |
| **Mean Duration–B** | 0.001 | 0.12 | 0.998 | 0.649 |
| **Mean Duration–C** | 0.11 | 0.06 | 0.656 | 0.828 |
| **Mean Duration–D** | 0.50 | -0.27 | 0.035 | 0.286 |
| **Time Coverage–A** | 0.28 | -0.18 | 0.252 | 0.476 |
| **Time Coverage–B** | -0.32 | 0.32 | 0.203 | 0.203 |
| **Time Coverage–C** | -0.16 | 0.26 | 0.540 | 0.296 |
| **Time Coverage–D** | 0.16 | -0.34 | 0.536 | 0.168 |

**Results**

## **Demographic and clinical variables**


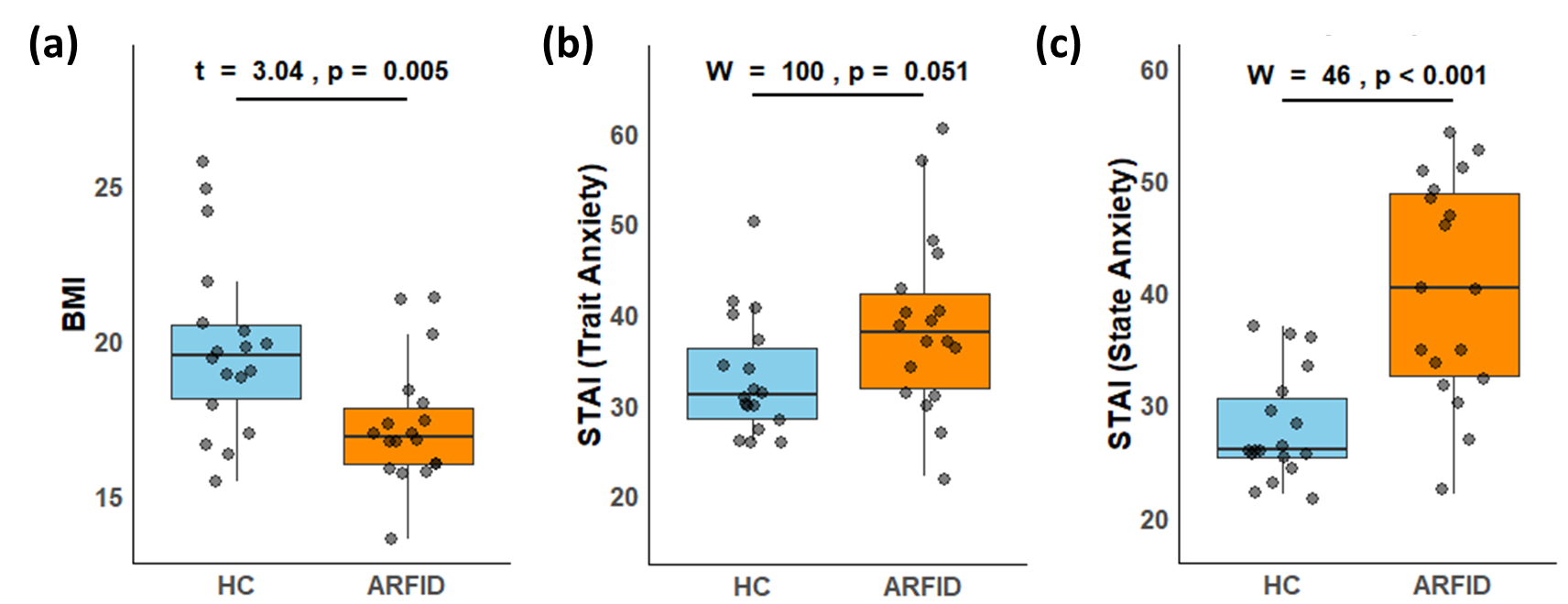


**Figure S2:** Group differences for demographic and clinical parameters: (a) BMI, (b) STAI trait, and (c) STAI state
